# Comparison of outcomes between immediate implant-based and autologous reconstruction: 15-year, single-center experience in a propensity score-matched Chinese cohort

**DOI:** 10.20892/j.issn.2095-3941.2021.0368

**Published:** 2021-12-01

**Authors:** Shanshan He, Bowen Ding, Gang Li, Yubei Huang, Chunyong Han, Jingyan Sun, Qingfeng Huang, Jing Liu, Zhuming Yin, Shu Wang, Jian Yin

**Affiliations:** 1 Department of Breast Reconstruction, Key Laboratory of Breast Cancer Prevention and Therapy, Tianjin Medical University, Ministry of Education, Sino-Russian Joint Research Center for Oncoplastic Breast Surgery, Tianjin Medical University Cancer Institute & Hospital, National Clinical Research Center for Cancer, Key Laboratory of Cancer Prevention and Therapy, Tianjin, Tianjin’s Clinical Research Center for Cancer, Tianjin 300060, China; 2 School of Pharmacy, University College London, London WC1N 1AX, UK; 3 Department of Cancer Epidemiology and Biostatistics, Tianjin Medical University Cancer Institute & Hospital, National Clinical Research Center for Cancer, Key Laboratory of Cancer Prevention and Therapy, Tianjin, Tianjin’s Clinical Research Center for Cancer, Tianjin 300060, China

**Keywords:** Oncological safety, immediate breast reconstruction, implant-based, autologous, Chinese, propensity-score matched

## Abstract

**Objective:**

The number of immediate breast reconstruction (IBR) procedures has been increasing in China. This study aimed to investigate the oncological safety of IBR, and to compare the survival and surgical outcomes between implant-based and autologous reconstruction.

**Methods:**

Data from patients diagnosed with invasive breast cancer who underwent immediate total breast reconstruction between 2001 and 2016 were retrospectively reviewed. Long-term breast cancer-specific survival (BCSS), disease-free survival (DFS), and locoregional recurrence-free survival (LRFS) were evaluated. Patient satisfaction with the breast was compared between the implant-based and autologous groups. BCSS, DFS, and LRFS were compared between groups after propensity score matching (PSM).

**Results:**

A total of 784 IBR procedures were identified, of which 584 were performed on patients with invasive breast cancer (implant-based, *n* = 288; autologous, *n* = 296). With a median follow-up of 71.3 months, the 10-year estimates of BCSS, DFS, and LRFS were 88.9% [95% confidence interval (CI) (85.1%–93.0%)], 79.6% [95% CI (74.7%–84.8%)], and 94.0% [95% CI (90.3%–97.8%)], respectively. A total of 124 patients completed the Breast-Q questionnaire, and no statistically significant differences were noted between groups (*P* = 0.823). After PSM with 27 variables, no statistically significant differences in BCSS, DFS, and LRFS were found between the implant-based (*n* = 177) and autologous (*n* = 177) groups. Further stratification according to staging, histological grade, lymph node status, and lymph-venous invasion status revealed no significant survival differences between groups.

**Conclusions:**

Both immediate implant-based and autologous reconstruction were reasonable choices with similar long-term oncological outcomes and patient-reported satisfaction among patients with invasive breast cancer in China.

## Introduction

According to the Global Cancer Incidence, Mortality and Prevalence (GLOBOCAN) 2020 database, female breast cancer has surpassed lung cancer and is the most commonly diagnosed cancer worldwide^1^. In the comprehensive modern treatment modality for female breast cancer, immediate breast reconstruction following total mastectomy has become a common procedure, because it has been shown to improve patient quality of life and psychosocial well-being^2,3^. However, scientific concerns have been raised suggesting that the reconstruction procedure, regardless of reconstruction type, contributes to hypoxia, provides a wound microenvironment, and adipose-derived stem cells, and leaves a dermal reservoir of cancer cells, all of which could potentially stimulate cancer recurrence^4^. Another concern is that immediate reconstruction may be associated with higher postoperative complications and could delay the initiation of subsequent adjuvant therapy, thus decreasing survival^5–7^. However, most clinical studies have not shown inferior oncological outcomes with respect to those of immediate breast reconstruction^8–11^ following mastectomy and mastectomy alone.

In China, there is a paucity of reports addressing oncological outcomes after immediate breast reconstruction. Given the growing trend favoring implant-based breast reconstruction^12–14^ and the new oncological issues with textured breast implants^15^, we sought to compare the long-term survival outcomes between implant and autologous reconstruction in a cohort in the immediate setting in the Chinese population.

## Materials and methods

### Study population and design

Data from patients who underwent immediate breast reconstruction between May 2001 and March 2016 were retrospectively collected from the breast reconstruction database of the National Clinical Research Center for Cancer of Tianjin Medical University Cancer Institute & Hospital (Tianjin, China). Patient characteristics, oncological features, treatment variables, and surgical outcomes were recorded. Oncological data, including survival status, locoregional recurrence, and distant metastasis, were also retrieved from the database. Patients were asked to complete the Breast-Q^16^ questionnaire during their clinical follow-up, and their most recent satisfaction with the breast(s) was recorded. Only patients diagnosed with invasive breast cancer were included in the study cohort. Patients diagnosed with stage IV disease or inflammatory breast cancer, and those who underwent wide local excision followed by partial breast reconstruction were excluded.

Patients were assigned to the implant-based or autologous groups on the basis of the reconstruction type. To recognize the potential for confounding at the patient, tumor, and comprehensive treatment levels, we first calculated a propensity score by using logit regression to predict the likelihood of the type of reconstruction according to patient and clinical characteristics. Propensity score matching (PSM) was then performed by using the nearest-neighbor method at a 1:1 ratio, with the caliper width set to 0.19. Standardized mean differences in the variables and density plot of the distribution of balance were reviewed both before and after matching^17–19^. Breast cancer-specific survival (BCSS), disease-free survival (DFS), and locoregional recurrence-free survival (LRFS) were compared between groups.

BCSS was defined as the time from the date of diagnosis to death due to breast cancer. Locoregional recurrence was defined as recurrence in the ipsilateral chest wall (skin, subcutaneous tissue, or muscle) supraclavicular/infraclavicular region, axilla, and/or internal mammary region. All cases of locoregional recurrence were confirmed pathologically. DFS was defined by the presence of locoregional recurrence or distant metastasis. All cases of distant metastases were confirmed with imaging [e.g., computed tomography (CT), positron emission tomography CT, bone scanning, magnetic resonance imaging, and/or ultrasound], or pathological biopsy.

This study was approved (Approval No. bc2021103) by the Ethical Committee and Institutional Review Board of the Tianjin Medical University Cancer Institute & Hospital and was conducted in accordance with the Declaration of Helsinki.

### Reconstruction techniques

Immediate breast reconstruction was performed after mastectomy. For implant-based reconstruction, a permanent textured breast implant (or tissue expander) was placed in the submuscular layer, with the inferolateral area covered by either the serratus anterior or latissimus dorsi. If a tissue expander was used, it was later changed to a permanent textured implant. For autologous reconstruction, either the pedicled (latissimus dorsi or transverse rectus abdominis myocutaneous flap) or the free deep inferior epigastric flap procedure was used.

### Statistical analysis

Categorical variables were compared with Pearson’s chi-squared test or Fisher’s exact test for expected frequencies <5. Continuous variables were compared with the *t*-test or Wilcoxon rank-sum test if the data did not meet the assumption of normality per the Shapiro–Wilk test. The BCSS, RFS, and LRFS for the study population were generated with the Kaplan-Meier method, and differences were compared with the log-rank test. If the 2 survival curves crossed each other, cross-points were analyzed, and this was followed by landmark analysis or a 2-stage procedure test to compare the differences^20,21^. All analyses were performed in R software version 4.0.2 (http://www.r-project.org); the main packages used included survival, tableone, cobalt, survminer, ComparisonSurv, TSHRC, and MatchIt. All statistical tests were performed at a 2-sided significance level of 0.05.

## Results

### Baseline patient characteristics and PSM

**Figure 1:**
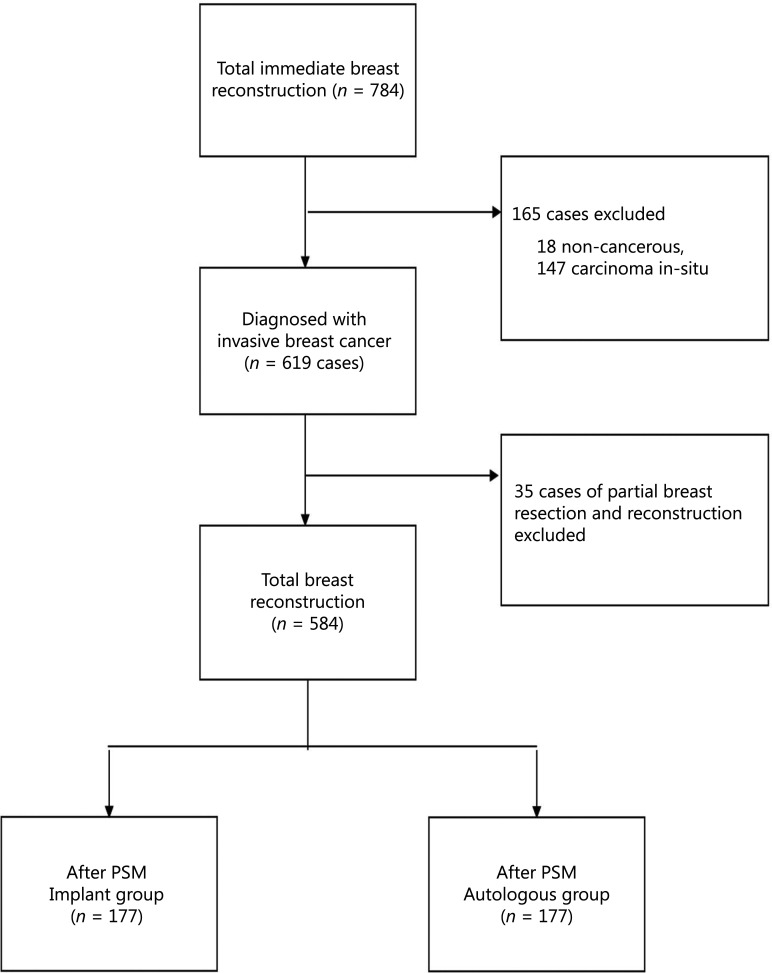
Flowchart for patient attrition. PSM, propensity score matching.

A total of 784 immediate breast reconstruction procedures performed during the study period were identified in the database. Among these, 574 patients diagnosed with invasive breast cancer with 584 sides of immediate reconstruction were eligible for analysis. A flow diagram illustrating patient inclusion is presented in **Figure 1**. Among the study cohort, 288 cases were implant based, and 296 were autologous based.

**Figure 2:**
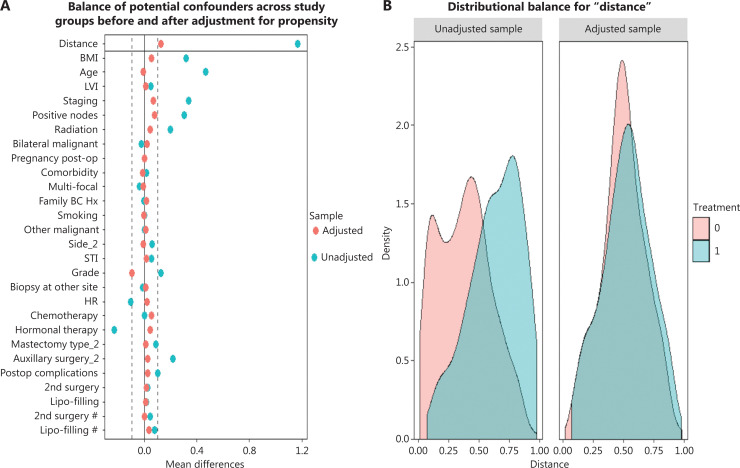
(A) Balance of the covariates used in generating propensity score matching before (blue) and after (red) adjustment. Dashed lines indicate acceptable limits for balance. (B) Density plot showing the distribution balance before and after propensity score matching between the implant (red) and autologous group (blue).

Baseline characteristics were compared between the implant-based and autologous groups. Patients undergoing autologous reconstruction were more likely to have soft-tissue invasion, unifocal breast cancer, late American Joint Committee on Cancer (AJCC) staging, hormone receptor (HR)-negativity, less nipple-sparing mastectomy, more comprehensive axillary lymph node dissection, more neo-adjuvant chemotherapy and postoperative radiation, and more postoperative complications (**Tables 1** and **2**). After PSM by 27 variables, each group had 177 cases remained (autologous *n* = 177, implant based *n* = 177) (**Figure 2A–B**). No significant differences were found between groups in terms of baseline characteristics (**Tables 1** and **2**).

**Table 1:** Baseline patient characteristics before and after propensity score matching (categorical variables)

Categorical variables	Before matching			After matching		
	Implant (*n* = 288) number (%)	Autologous (*n* = 296) number (%)	*P* value	Implant (*n* = 177) number (%)	Autologous (*n* = 177) number (%)	*P* value
Fx BC history			0.92^#^			0.84^#^
No	264 (45.2)	273 (46.7)		165 (46.6)	163 (46.0)	
Yes	24 (4.1)	23 (3.9)		12 (3.4)	14 (4.0)	
Smoking status			0.62^$^			1.00^$^
No	286 (49.0)	295 (50.5)		175 (49.4)	176 (49.7)	
Yes	2 (0.3)	1 (0.2)		2 (0.6)	1 (0.3)	
Bilateral malignant tumor			0.247^#^			0.64^#^
No	268 (45.9)	283 (48.5)		169 (47.7)	166 (46.9)	
Yes	20 (3.4)	13 (2.2)		8 (2.3)	11 (3.1)	
Pregnancy post-op			0.62^$^			1.00^#^
No	286 (49.0)	295 (50.5)		177 (50.0)	177 (50.0)	
Yes	2 (0.3)	1 (0.2)		0 (0)	0 (0)	
Side			0.21^#^			0.91^#^
Left	154 (26.4)	142 (24.3)		87 (24.6)	89 (25.1)	
Right	134 (22.9)	154 (26.4)		90 (25.4)	88 (24.9)	
LVI			0.08^#^			1.00^#^
No	268 (45.9)	262 (44.9)		162 (45.8)	161 (45.5)	
Yes	20 (3.4)	34 (5.8)		15 (4.2)	16 (4.5)	
STI			0.02^#,^*			0.84^#^
No	275 (47.1)	267 (45.7)		164 (46.3)	162 (45.8)	
Yes	13 (2.2)	29 (5.0)		13 (3.7)	15 (4.2)	
Grade			0.25^#^			0.55^#^
I	11 (1.9)	10 (1.7)		5 (1.4)	8 (2.3)	
II	249 (42.6)	244 (41.8)		154 (43.5)	155 (43.8)	
III	28 (4.8)	42 (7.2)		18 (5.1)	14 (4.0)	
Multi-focal			0.04^#^			0.81^#^
No	265 (45.4)	285 (48.8)		167 (47.2)	169 (47.7)	
Yes	23 (3.9)	11 (1.9)		10 (2.8)	8 (2.3)	
AJCC stage			<0.001^#^			0.73^#^
I	113 (19.3)	79 (13.5)		60 (16.9)	53 (15.0)	
II	150 (25.7)	162 (27.7)		93 (26.3)	99 (28.0)	
III	25 (4.3)	55 (9.4)		24 (6.8)	25 (7.1)	
HR status			0.003^#^			0.79^#^
Negative	49 (8.4)	82 (14.0)		36 (10.2)	33 (9.3)	
Positive	239 (40.9)	214 (36.6)		141 (39.8)	144 (40.7)	
Chemotherapy			0.003^#^			0.18^#^
None	16 (2.7)	4 (0.7)		10 (2.8)	3 (0.8)	
Neoadjuvant	21 (3.6)	41 (7.0)		18 (5.1)	23 (6.5)	
Adjuvant	216 (37.0)	221 (37.8)		127 (35.9)	133 (37.6)	
Unknown	35 (6.0)	30 (5.1)		22 (6.2)	18 (5.1)	
Radiation			<0.0001^#,^*			0.46^#^
No	239 (40.9)	188 (32.2)		137 (38.7)	130 (36.7)	
Yes	49 (8.4)	108 (18.5)		40 (11.3)	47 (13.3)	
Hormonal therapy			0.006^#,^*			0.89^#^
No	58 (9.9)	94 (16.1)		44 (12.4)	40 (11.3)	
Yes	220 (37.7)	193 (33.0)		126 (35.6)	130 (36.7)	
Unknown	10 (1.7)	9 (1.5)		7 (2.0)	9 (2.0)	
Breast surgery type			0.04^#,^*			1.00^#^
NSM	117 (20.0)	95 (16.3)		65 (18.4)	64 (18.1)	
SSM	171 (29.3)	201 (34.4)		112 (31.6)	113 (31.9)	
Axillary surgery			<0.0001^#,^*			0.55^#^
SLNB	73 (12.5)	12 (2.1)		16 (4.5)	12 (3.4)	
ALND	215 (36.8)	284 (48.6)		161 (45.5)	165 (46.6)	
Post-op complications			<0.01^#,^*			0.62^#^
No	260 (44.5)	238 (40.8)		158 (44.6)	154 (43.5)	
Yes	28 (4.8)	58 (9.9)		19 (5.4)	23 (6.5)	
Secondary surgery			0.43^#^			0.84^#^
No	267 (45.7)	268 (45.9)		164 (46.3)	162 (45.8)	
Yes	21 (3.6)	28 (4.8)		13 (3.7)	15 (4.2)	
Lipo-filling			0.30^#^			1.00^$^
No	286 (49.0)	325 (49.7)		175 (49.4)	174 (49.2)	
Yes	2 (0.3)	6 (1.0)		2 (0.6)	3 (0.8)	

Secondary surgery refers to revision or salvage surgery that was not associated with tumor relapse. Abbreviations: Fx BC history, family breast cancer history; LVI, lymph-vascular invasion; STI, soft tissue invasion; HR, hormonal receptor; NSM, nipple sparing mastectomy; SSM, skin sparing mastectomy; SLNB, sentinel lymph node biopsy; ALND, axillary lymph node dissection. ^#^Pearson’s chi-square test. ^$^Fisher’s exact test. *P < 0.05.

**Table 2:** Baseline patient characteristics before and after propensity score matching (numerical variables)

Numerical variables	Before matching			After matching		
	Implant (*n* = 288) mean ± SD	Autologous (*n* = 331) mean ± SD	*P* value	Implant (*n* = 183) mean ± SD	Autologous (*n* = 183) mean ± SD	*P* value
Age (years)	38.4 ± 0.5	41.7 ± 0.4	<0.0001^†,^*	39.6 ± 0.6	39.6 ± 0.5	0.92^φ^
BMI (kg/m^2^)	22.4 ± 0.1	23.3 ± 0.2	<0.0001^†,^*	22.8 ± 0.2	22.9 ± 0.2	0.61^†^
Positive nodes	0.9 ± 0.1	2.5 ± 0.3	<0.0001^†,^*	1.4 ± 0.2	1.8 ± 0.3	0.47^†^
Total nodes	16.2 ± 0.5	20.0 ± 0.4	<0.0001^†,^*	18.1 ± 0.6	19.0 ± 0.5	0.15^†^
Number of 2^nd^ surgery	0.1 ± 0.0	0.1 ± 0.0	0.49^†^	0.1 ± 0.0	0.1 ± 0.0	0.83^†^
Number of lipo-filling	0.0 ± 0.0	0.0 ± 0.0	0.17^†^	0.0 ± 0.0	0.0 ± 0.0	0.66^†^

Secondary surgery refers to revision or salvage surgery that was not associated with tumor relapse. ^†^Wilcoxon rank sum test. ^φ^Welch 2-sample t test. *P < 0.05.

### Analysis of BCSS, DFS, and LRFS in the study population

**Figure 3:**
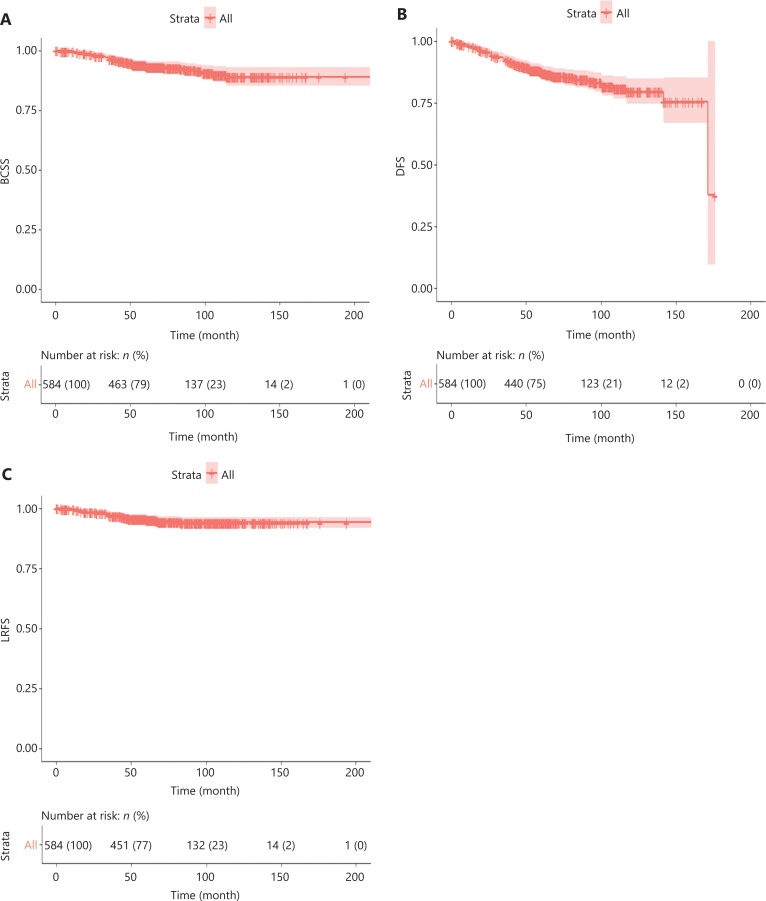
(A) Breast cancer-specific survival, (B) disease-free survival, and (C) locoregional recurrence-free survival for the entire invasive breast cancer cohort with immediate reconstruction before propensity score matching.

The median follow-up for all 584 cases was 71.3 months [interquartile range (IQR) 54.1–101.5]. Kaplan-Meier method estimates of the 5-year BCSS, DFS, and LRFS were 93.8% [95% confidence interval (CI) 81.8%–95.9%], 87.7% (95% CI 84.9%–90.5%), and 96.6% (95% CI 94.5%–98.8%), respectively. The 10-year estimates for BCSS, DFS, and LRFS were 88.9% (95% CI 85.1%–93.0%), 79.6% (95% CI 74.7%–84.8%), and 94.0% (95% CI 90.3%–97.8%), respectively (**Figure 3A–C**).

### Analysis of patient-reported outcomes in the study population

To investigate patient-reported outcomes (PROs) of immediate reconstruction for invasive breast cancer, data on 124 patients who had completed the Breast-Q questionnaire postoperative reconstruction module during the latest clinical follow-up were collected. Sixty-four patients were in the implant group, and 60 were in the autologous group. With a median PRO follow-up of 61.0 months (IQR 53.0–67.5), the mean Q score for satisfaction with the breast in the implant group was 71.20 ± 2.75. With a median PRO follow-up of 85.5 months (IQR 65.0–105.0), the mean Q score for satisfaction with the breast in the autologous group was 70.12 ± 2.45. No statistically significant differences were observed between groups (*P* = 0.823).

### Absence of effects of reconstruction type on BCSS, DFS, and LRFS after PSM

After PSM, the median follow-up for the implant group and autologous group was 68.37 months (IQR 53.52–92.81) and 79.05 months (IQR 58.22–109.70), respectively. No significant statistical differences were observed between the implant and autologous groups in terms of breast cancer-related death (2.3% *vs.* 4.5%; *P* = 0.84), locoregional recurrence (1.7% *vs*. 2.8%; *P* = 0.44), or distant metastasis (5.9% *vs*. 6.8%; *P* = 0.75).

**Figure 4:**
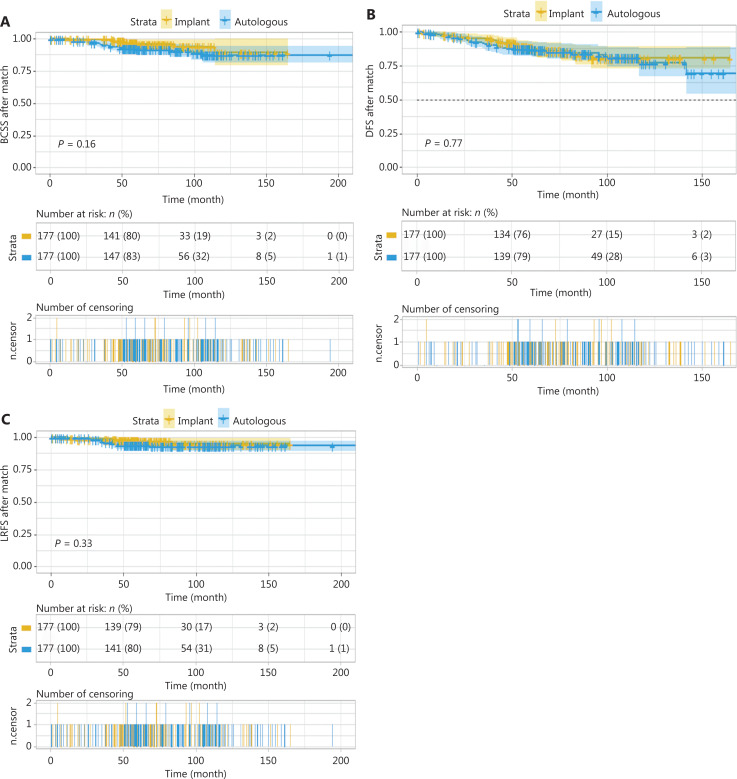
(A) Breast cancer-specific survival, (B) disease-free survival, and (C) locoregional recurrence-free survival between the immediate implant-based and autologous breast reconstruction for patients with invasive breast cancer after propensity score matching. No significant differences were found between groups.

The 5-year BCSS for the implant and autologous groups was 96.7% (95% CI 93.8%–99.6%) and 92.7% (95% CI 88.8%–96.8%), respectively, and the 10-year BCSS was 89.0% (95% CI 79.4%–99.8%) and 87.3% (95% CI 81.1%–94.1%), respectively. No statistically significant difference (*P* = 0.16) was observed between the groups (**Figure 4A**).

For the implant group, the 5- and 10-year DFS rates were 87.9% (95% CI 82.8%–93.2%) and 80.5% (95% CI 72.8%–88.9%), respectively. For the autologous group, the 5- and 10-year DFS rates were 87.3% (95% CI 82.3%–92.5%) and 77.0% (95% CI 67.3%–88.0%), respectively, and no significant differences were found between groups (*P* = 0.77). Because the curves crossed at multiple time points with the log-rank test, a 2-stage procedure was used, and the difference was not found to be statistically significant between groups (*P* = 0.60) (**Figure 4B**).

For the implant group, the 5-and 10-year LRFS rates were 97.6% (95% CI 95.3%–100.0%) and 94.7% (95% CI 90.1%–99.6%), respectively. For the autologous group, the 5- and 10-year LRFS rates were 94.3% (95% CI 90.7%–98.0%) and 93.2% (95% CI 89.2%–97.5%), respectively, and no significant differences were found between groups (*P* = 0.33). Because the curves crossed at 27.60 months, further landmark analysis was performed and revealed no significant differences between groups (*P* = 0.30) (**Figure 4C**).

### Absence of significant survival differences between reconstruction groups after stratification by stage, histological grade, lymph node status, and lymph-venous invasion status

Survival differences between the reconstruction groups were further compared after stratification according to the stage, histological grading, lymph node positivity, and lymph-venous invasion status. No statistically significant differences were found in BCSS, DFS, or LRFS in patients with advanced stage III (*P* = 0.12, *P* = 0.56, and *P* = 0.17, respectively), high histological grade (*P* = 0.14, *P* = 0.77, *P* = 0.31), positive lymph node (*P* = 0.07, *P* = 0.62, *P* = 0.09), and lymph-venous invasive breast cancer (*P* = 0.68, *P* = 0.32, *P* = 0.19) (**Supplementary Figure S1A–L**).

## Discussion

Immediate breast reconstruction has become an important component of comprehensive treatment for female breast cancer patients. The oncological safety of either autologous or implant-based breast reconstruction has been reported by many cancer centers worldwide^11,22–24^. However, reports documenting the oncological outcomes between implant-based and autologous reconstructions are scarce. To our knowledge, this study is the first to report the long-term oncological safety of immediate breast reconstruction and to compare the survival differences between reconstruction types in a matched invasive breast cancer cohort in Chinese population.

In our study, with a median follow-up of 71.3 months, the 5-year estimates of BCSS for the entire immediate reconstruction cohort reached 93.8%, which was comparable to the 94% overall survival rate in a stage 0–III cohort reported by Siotos et al.^11^ The 5-year LRFS rate estimate in our study was 96.6%, which was similar to the 96.0% for mastectomy alone and 96.0% for mastectomy and reconstruction cohorts of breast cancer patients reported by the Fudan University Shanghai Cancer Center^25^. Park et al.^26^ have reported a 5-year LRFS rate of 96.2% and a DFS rate of 92% in their immediate reconstruction for invasive breast cancer cohort. Our study yielded a slightly lower DFS rate. Our longer follow-up duration and higher proportion of patients with advanced stage, neoadjuvant chemotherapy, and radiation therapy might have contributed to this difference.

Although the use of implant-based breast reconstruction has been increasing worldwide, specific types of breast implants have been established to be associated with anaplastic large cell lymphoma, possibly because of the chronic inflammatory environment that they create^27^. Furthermore, scientific studies have provided ample evidence supporting an association between an inflammatory environment and breast cancer recurrence^28^. In a retrospective study, Lee et al.^29^ reported lower DFS rates with textured implant reconstruction compared with smooth implant reconstruction, particularly in the advanced stage and invasive breast cancer subgroups, thus bringing the safety concerns of breast implants—particularly textured—into the spotlight. In our cohort of patients with invasive breast cancer, all the implants were textured. After balancing of 27 potential confounding variables with PSM, no statistically significant differences in BCSS, DFS, and LRFS were found between the implant-based and autologous groups. Our results were consistent with those reported in an earlier study by Shao et al.^30^, which has reported similar BCSS and overall survival between immediate implant reconstruction and autologous reconstruction in patients with invasive ductal carcinoma breast cancer, on the basis of analysis of data from the SEER database in the United States population. Our study further strengthened the results by providing oncological outcomes for local recurrence and metastasis in terms of LRFS and DFS. After stratification according to AJCC staging, no significant statistical differences in survival outcomes were observed between groups among advanced-stage patients. Our results suggest that immediate textured implant-based reconstruction, as compared with autologous reconstruction, is an oncologically safe procedure, thus providing reassurance that the type of reconstruction did not affect survival outcomes for patients with invasive breast cancer.

High histological grade, advanced stage, and lymph-venous invasion^31–33^ are well-known prognostic factors for breast cancer. Ha et al.^34^ have reported lower DFS but similar LRFS in flap reconstruction compared with implant reconstruction in patients with high histological grade breast cancer, thus suggesting that the longer surgical stress duration involved in autologous reconstruction may play a role in distant tumor metastasis. However, the subgroup analysis was based on a small sample size (approximately 150 patients) and might have been subject to bias. In our study cohort with a longer follow-up duration, we did not observe adverse effects of autologous reconstruction on distant metastasis in patients with a high histological grade, positive lymph node status, and positive lymph-venous invasion. We believe that the oncological outcomes of breast cancer treatment largely rely on the nature of the cancer cells and the proper delivery of comprehensive treatment. The choice of reconstruction type was more relevant to the considerations of postoperative aesthetics, and the timely and adequate delivery of adjuvant therapies (e.g., radiation), if needed.

The ultimate goal of immediate breast reconstruction is to improve patient quality of life. Development of the Breast-Q has introduced a standardized evaluation system to measure PRO and has been widely used worldwide. Several studies^35,36^ have reported higher satisfaction with the breast in the autologous group than in the implant group among patients undergoing immediate breast reconstruction, particularly when postoperative radiation was involved^37^. However, in our study with a median 5- and 7-year follow-up, no significant differences were found between the implant and autologous groups. A higher proportion of patients undergoing radiation in the autologous group might have diminished the differences observed in other studies. Because of the retrospective design of our study, we were unable to compare the PRO results in the PSM cohorts; therefore, further studies addressing this issue are warranted.

Our study has several limitations. Owing to the adverse effects of radiation therapy on breast implants, surgeons performed more autologous reconstructions in advanced-stage patients. Other oncological and socioeconomic factors might also have contributed to the surgeons’ decisions regarding reconstruction type. We attempted to mitigate this selection bias by using PSM; however, we acknowledge that balanced, but reduced, samples may not represent the entire population. Given the retrospective nature of this study, we were unable to collect data on the status of several important molecular markers, such as Her-2, Ki-67, and p53, in the entire study population; therefore, we excluded these markers from our analyses. Further prospective studies with detailed molecular and gene testing results would consolidate our initial findings.

## Conclusions

In our cohort of Chinese patients with invasive breast cancer who underwent immediate breast reconstruction, no significant differences in survival were found between the textured implant-based and autologous groups. The choice of reconstruction type did not affect the long-term oncological outcomes of patients with breast cancer.
