# Supplementary material for: Comparison of outcomes between immediate implant-based and autologous reconstruction: 15-year, single-center experience in a propensity score-matched Chinese cohort
Source: Cancer Biol Med. 2021 Dec 1;19(9):1410–21. doi: 10.20892/j.issn.2095-3941.2021.0368 (PMC9500225; doi:10.20892/j.issn.2095-3941.2021.0368)
Supplement: Supplementary file 1 [file cbm-19-1410-s001.pdf]

Supplementary material

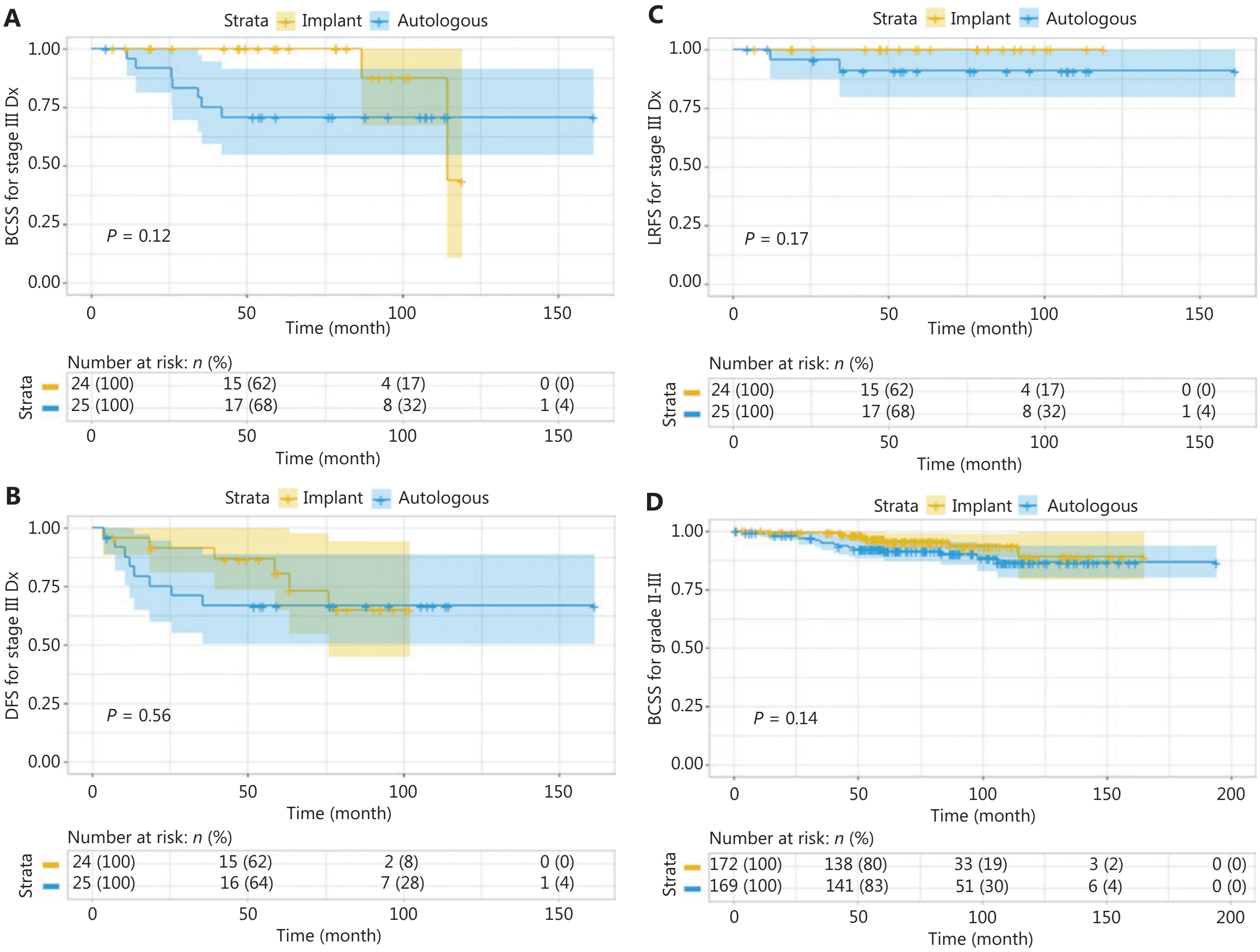

Figure S1 Continued

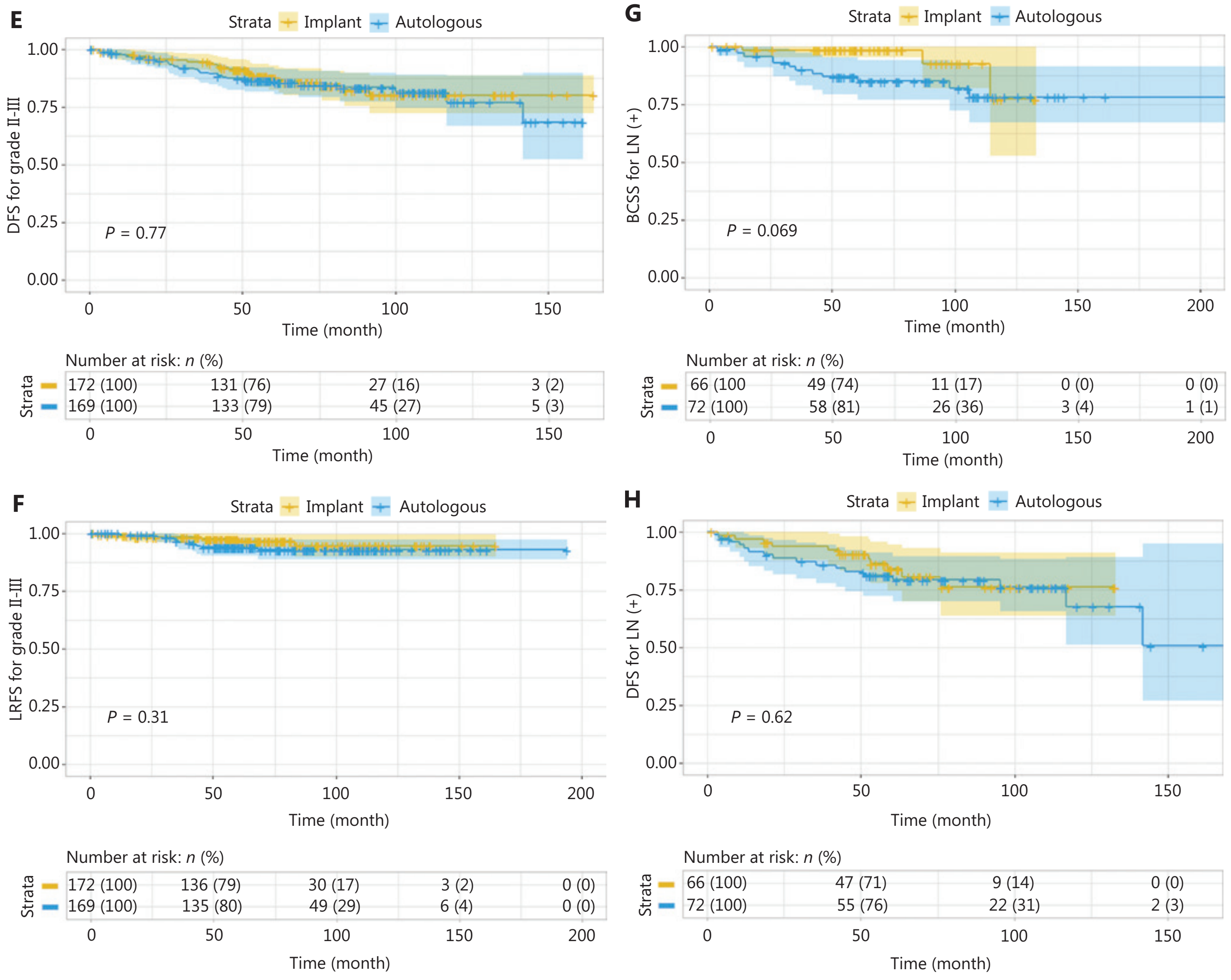

Figure S1 Continued

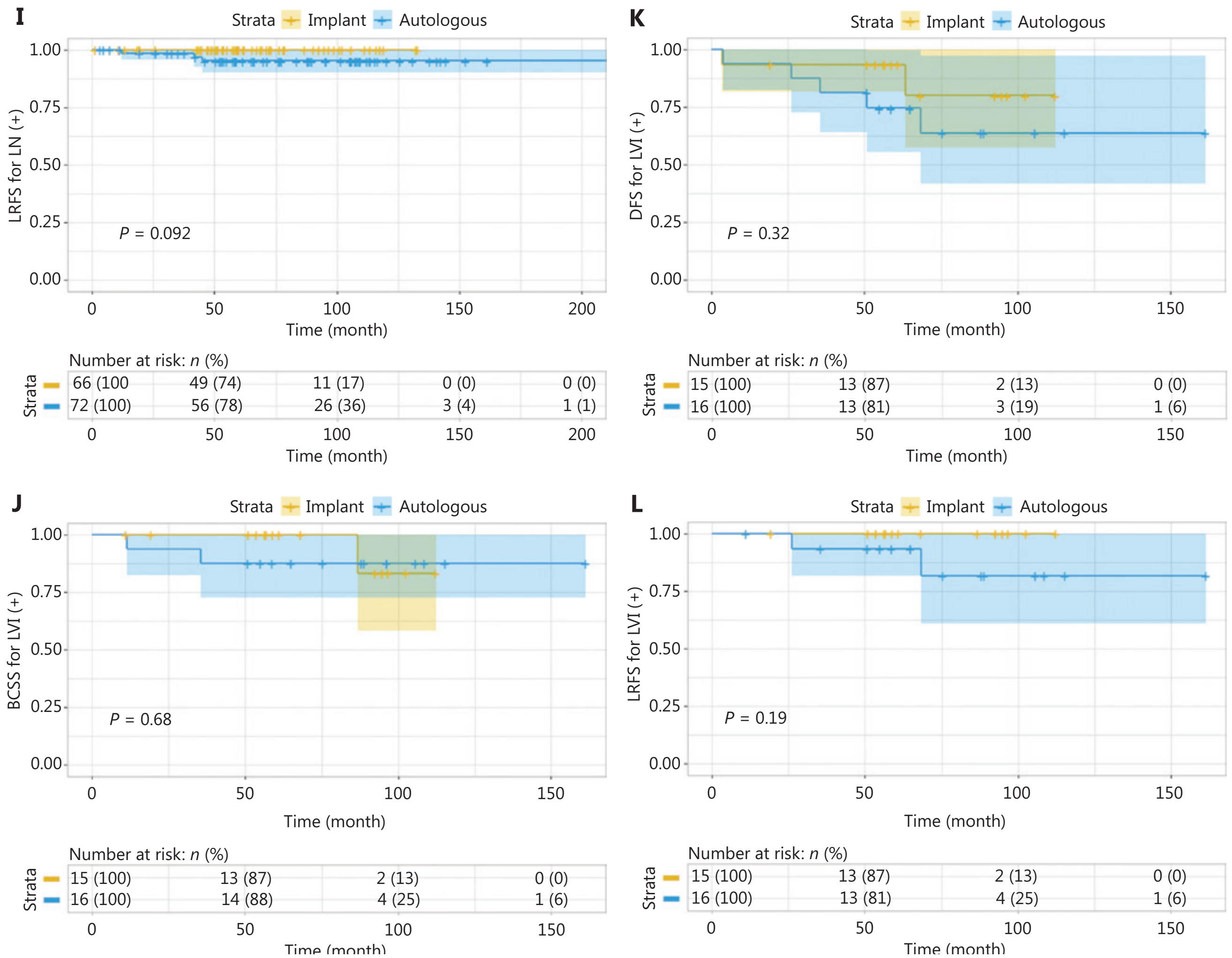

Figure S1 Breast cancer-specific survival (BCSS), disease-free survival (DFS), and locoregional recurrence-free survival (LRFS) after stratification in patients with (A–C) advanced stage, (D–F) high histological grade, (G–I) positive lymph node, and (J–L) positive lymph-venous invasion in the propensity score matched cohort.
